# Primary omental pregnancy in a subseptate uterus: A novel case report and literature review

**DOI:** 10.1016/j.amsu.2022.104924

**Published:** 2022-11-14

**Authors:** Nuha Fouad Abed Althagafi, Maad Galal, Saleha Abdul Rab, Anas Alkhudari, Hiba Muhammad Raheel

**Affiliations:** a Department of Obstetrics and Gynecology, King Abdulaziz Medical City, Riyadh, Saudi Arabia; b College of Medicine, Alfaisal University, Riyadh, Saudi Arabia

**Keywords:** Primary omental pregnancy, Ectopic pregnancy, Septate uterus, Partial omentectomy, Miscarriage, Case report

## Abstract

**Introduction and importance:**

Ectopic pregnancy is defined as a pregnancy in which the fertilized ovum implants itself in a location other than the uterine endometrium. Abdominal ectopic pregnancies involve the implantation and development of the embryo within the peritoneal cavity. Primary omental pregnancies are the rarest form of abdominal pregnancy and possibly the rarest extrauterine gestation.

**Case presentation:**

We report the first case of a primary omental pregnancy in a subseptate uterus in literature. Our patient, a 33-year-old female, G8P4+3, presented with nausea, severe abdominal pain, and vaginal spotting at 6 weeks’ gestational age. She had mild tenderness below the umbilicus, with positive cervical and right adnexal tenderness. 2D-ultrasound revealed a subseptate uterus, normal ovaries and fallopian tubes, absence of a gestational sac, and a 4x3x2.5 cm mass in the right adnexa. A mini-laparotomy was performed due to suspicion of ruptured tubal pregnancy, revealing a primary omental pregnancy which was managed via partial omentectomy.

**Clinical discussion:**

Ectopic pregnancies have ambiguous presentations, however correct diagnosis and management is crucial to prevent complications. A high index of suspicion must be exercised to make an accurate diagnosis of primary omental pregnancy. A subseptate uterus is a subtype of the most common uterine anomaly and should be investigated via 3D-ultrasound and magnetic resonance imaging as it causes increased risk of primary omental implantation.

**Conclusion:**

Correct identification of subseptate or septate uteri is vital. Greater research is needed to elucidate the association between septate or subseptate uteri and ectopic pregnancy, particularly primary omental pregnancy.

## Highlights

• Primary omental pregnancies are the rarest form of abdominal pregnancy and possibly the rarest extrauterine gestation.
• A high index of suspicion must be maintained to correctly diagnose primary omental pregnancies.
• Subseptate uteri are subtypes of septate uteri; this abnormality warrants further investigation with 3D-US and MRI.
• More research is needed to determine how subseptate uteri contribute to ectopic–particularly primary omental–pregnancies.

## 1 Introduction

Ectopic pregnancy is defined as a pregnancy in which the fertilized ovum implants itself in a location other than the uterine endometrium, i.e., an extra-uterine implantation [1]. Due to their ambiguous and varying clinical presentation, ectopic pregnancies pose a diagnostic challenge for the practicing gynecologist. Early diagnosis is essential to prevent morbidity, as complicated ectopic pregnancies may lead to severe bleeding or rupture (e.g., tubal rupture), and are considered a gynecological emergency [2]. Notable risk factors for ectopic pregnancy include a history of pelvic inflammatory disease (PID), previous ectopic pregnancy, previous spontaneous abortion, and anatomical uterine abnormalities such as a bicornuate or septate uterus [3,4]. These risk factors must be carefully identified and investigated.

Abdominal ectopic pregnancies represent 1.3% of ectopic pregnancies and involve the implantation and development of the embryo in the omentum, organs, or blood vessels within the peritoneal cavity [5]. Omental pregnancy can be of two forms: primary or secondary. Primary omental pregnancy is defined by implantation of an ovum directly into the abdominal cavity leaving the fallopian tubes and ovaries intact [6]. Secondary omental pregnancy refers to an extrauterine pregnancy that aborts or ruptures, followed by the embryo reimplanting in the abdomen [6]. Primary omental pregnancy is the rarest form of abdominal pregnancy [7], and is believed to also be the rarest form of extrauterine gestation. Herein, we report our experience in managing a patient with a primary omental pregnancy in a patient with a subseptate uterus. This case report is reported in line with the SCARE criteria [8].

## 2 Case Presentation

A 33-year-old Saudi female, G8P4+3, presented to our hospital after 6 weeks of amenorrhea (last menstrual period 6 weeks ago), complaining of nausea, severe abdominal pain, and minimal vaginal spotting for the past two days. Her history was significant for two miscarriages, PID, and an ectopic right tubal pregnancy, managed by laparoscopic partial right salpingectomy. Aside from that, she had regular menstrual history. She was admitted for management.

On admission, the patient was vitally stable and afebrile. On physical examination, mild tenderness was observed below the umbilicus, extending to the right iliac fossa. Bimanual vaginal examination revealed positive cervical and right adnexal tenderness. Laboratory investigations revealed a hemoglobin level of 11.8 g/dL and a leukocyte count of 8.5 × 10^9^/L. Her serum β-human chorionic gonadotropin (β-HCG) level was 250 IU/L. 2D transvaginal ultrasound showed homogenous endometrium with a thickness of 5 mm, with no evidence of a yolk sac or gestational sac in the uterine cavity, normal ovaries, and evidence of a subseptate uterus that we were unaware of previously. In the right adnexa, superior to the right ovary, a heterogeneous vascular mass of size 4x3x2.5 cm was identified. A moderate amount of free fluid was seen in the left adnexa and the pouch of Douglas.

**Fig. 1:**
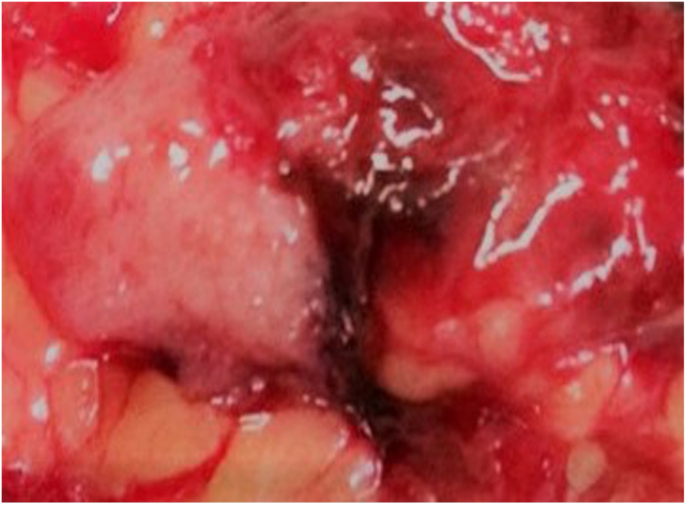
Intraoperative photo taken at the time of mini-laparotomy, showing primary omental pregnancy.

A mini-laparotomy incision was made, based on the provisional diagnosis of a ruptured tubal pregnancy. A nodular lesion was found, measuring 4x3x2.5 cm, invading the infracolic omentum, superior to the right adnexa. Fallopian tubes and ovaries were intact bilaterally, no uterine-peritoneal fistula was seen, and there were no adhesions between the nodular lesion and the uterus or adnexa, thereby meeting the diagnosis of primary omental pregnancy (Fig. 1). Additionally, there was no indication of adnexal origin. The mass was removed via partial omentectomy. About 300 cc of free clotted blood was evacuated from the pouch of Douglas.

**Fig. 2:**
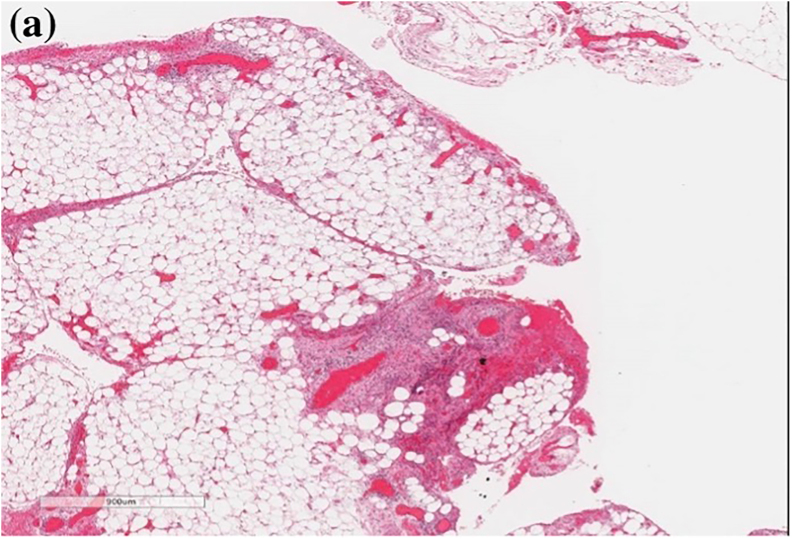

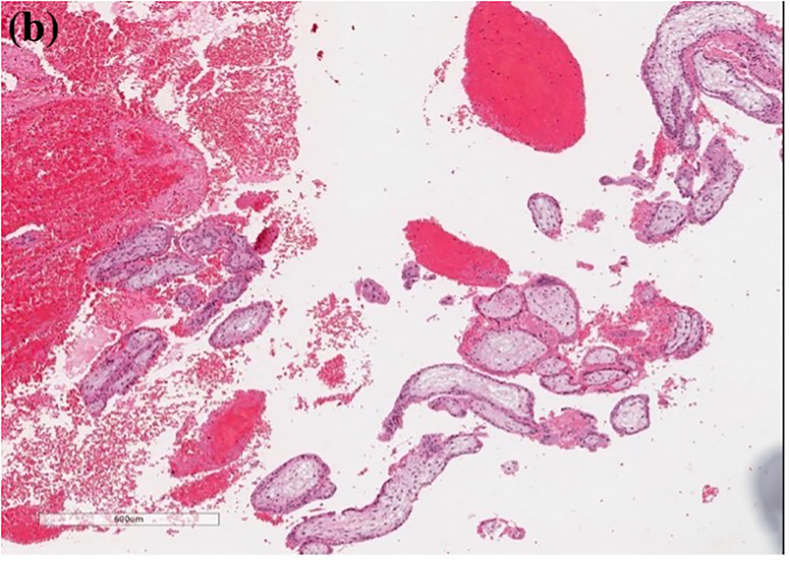
Omental fibro-adipose tissue seen on H&E staining, with hemorrhagic changes and mesothelial reactive hyperplasia secondary to the abdominal implantation of the trophoblastic tissue **(a)** at 40x magnification, and **(b)** at 100x magnification.

**Fig. 3:**
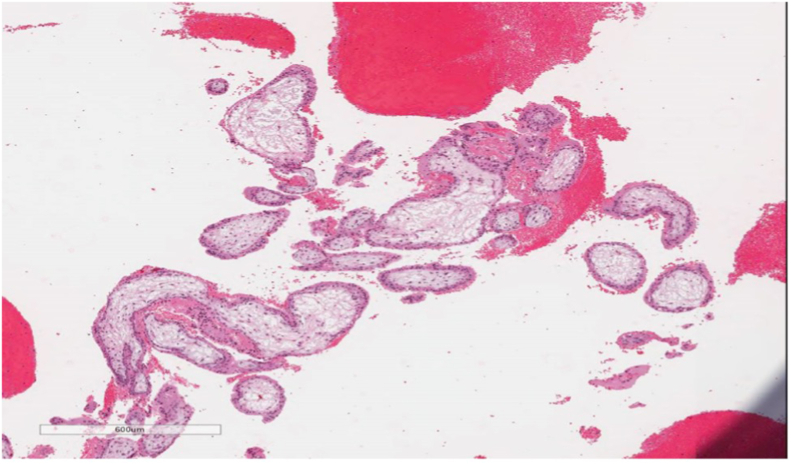
Light microscopic image at 100x magnification, showing the immature chorionic villi admixed with blood on H&E staining. Morphologically, the chorionic villi correspond with the first trimester of pregnancy.

Histopathology (Fig. 2, Fig. 3) revealed chorionic villi admixed with blood clots and fibro-adipose tissue, confirming primary omental pregnancy. The postoperative period was unremarkable. The patient was followed up for five years after the procedure to avoid future complications; during this time, she had one normal vaginal delivery, followed by an abortion at a gestational age of 8 weeks. The patient has been using contraception for the last two years.

## 3 Discussion

Ectopic pregnancy is an extrauterine pregnancy where the fertilized embryo implants itself in a location other than the uterine wall [1]. While uncomplicated ectopic pregnancies may resolve spontaneously, a complicated ectopic pregnancy can rupture and present acutely with severe lower abdominal pain, massive blood loss, and shock, and can be life-threatening [2]. Ruptured ectopic pregnancies account for nearly 3% of all pregnancy-related deaths [3], making an early diagnosis of ectopic pregnancy critical to avoid mortality.

Abdominal pregnancy have a reported incidence of 1:10,000 to 1:30,000 [5]. Omental pregnancy is the rarest form of abdominal pregnancy and can be of two types: (1) primary, which occurs when implantation of the ovum happens directly in the greater omentum [6]; and (2) secondary, which occurs following the rupture or abortion of an extrauterine pregnancy (usually tubal), after which the embryo implants in the abdomen [6]. A review of abdominal pregnancies found that only 11% were diagnosed preoperatively [9], therefore gynecologists must maintain a high index of suspicion to make the correct diagnosis [10].

While there is no consensus on the diagnosis of a primary omental pregnancy, the Studdiford criteria is frequently used which includes: (1) both ovaries and fallopian tubes must be normal, (2) there must be no uterine-peritoneal fistula, (3) pregnancy must be present only in the abdominal cavity, and (4) tubal pregnancy must be excluded [11]. These findings, combined with ultrasonographic, histopathological, and surgical findings, confirm the diagnosis. After a review of literature, we identified several cases of omental pregnancy, but none that discuss a subseptate (or partial septate) uterus.

As per the European Society of Human Reproduction and Embryology (ESHRE), a septate uterus is defined as a uterus with a division of the uterine cavity; a subseptate uterus refers to the presence of only a partial division in the uterus [12]. While the exact prevalence of subseptate uteri is unknown, septate uteri are the most common uterine anomaly and accounts for 35% of all uterine abnormalities [13]. Both septate and subseptate uteri put women of reproductive age at increased risk of subfertility, recurrent miscarriage, and premature delivery [13] – making them vital anomalies to rule out at the first incidence of miscarriage or ectopic pregnancy. However, they are easily recognized on standard 2D-ultrasound. Many report that 3D-ultrasound or magnetic resonance imaging (MRI) are strikingly more accurate for the diagnosis of a septate or subseptate uterus and should be performed in patients with recurrent miscarriage [14,15].

In this article, we report a patient who presented with severe abdominal pain, nausea, and vaginal spotting. She had a history of ectopic risk factors, including PID, two miscarriages, prior ectopic pregnancy, and partial right salpingectomy. A 2D transvaginal ultrasound revealed a subseptate uterus, not noted previously in this patient. A mini-laparotomy was performed, and a primary omental pregnancy was discovered and removed via partial omentectomy. To our knowledge, we are the first to report a case of primary omental pregnancy in a patient with a subseptate uterus.

It is unclear however, how a subseptate uterus may have contributed to the primary omental pregnancy. Previous reports of ectopic pregnancies in septate or subseptate uteri are limited and describe interstitial or cornual pregnancies [16,17], but none in the abdomen. For the sake of illustration, a report by Shehata et al. is worth discussion, where a primary abdominal pregnancy was discovered located on the peritoneum in a patient with a bicornuate uterus [18]. Several authors report that the distinction between bicornuate and septate uteri is ambiguous and sometimes inaccurate [19,20]. In this article, the gestational sac was implanted on the peritoneum between the two horns of uterus, illustrating that perhaps a malformed uterus, particularly of the bicornuate or septate type, increases the risk of abdominal implantation [18].

Secondly, it is worth noting that in previous reports regarding ectopic pregnancies in septate uteri, after 2D-ultrasound revealed an empty uterine cavity and/or an eccentrically located gestational sac, a 3D-ultrasound and MRI were performed immediately to confirm the diagnosis of the septate uterus as well as locate the pregnancy [16]. However, in our patient, a 3D-ultrasound and MRI were not performed immediately. Given that our case was a primary omental pregnancy, it is unknown whether further investigation of the subseptate uterus would have aided the diagnosis and management of this patient.

Thirdly, while a primary omental pregnancy in a patient with a subseptate or septate uterus has not been discussed in literature, similar cases exist regarding normal uteri. Several authors report primary omental pregnancies which, like our case, were presumed to be ruptured ectopic pregnancies, and were ultimately managed via partial omentectomy [10,21]. Non-emergent cases of primary omental pregnancy have also been reported, managed via laparoscopic partial omentectomy [22].

Given that an ectopic pregnancy, can be life-threating and requires invasive surgery, it is crucial to correctly identify risk factors in a patient with such history. An earlier diagnosis of a subseptate uterus would allow patients to undergo septoplasty to correct the anomaly to improve pregnancy outcome [23].

## 4 Conclusion

Primary omental pregnancies present a diagnostic challenge due to their often-ambiguous presentation. Correctly identifying risk factors in patients with recurrent miscarriages and/or a history of ectopic pregnancy is crucial. We report the first known case of primary omental pregnancy in a patient with a subseptate uterus. There are two takeaway points from this case report: (1) that the prevalence of primary omental pregnancy is exceedingly rare, and a high index of suspicion must be exercised by clinicians to make an accurate diagnosis before a critical situation arises; and (2) that a subseptate or septate uterus is a significant uterine anomaly to be further investigated via 3D-ultrasound or MRI in all patients with poor pregnancy history, or where evidence of a septate uterus is noted on 2D-ultrasound. We urge that future research be conducted to elucidate the association between such uterine anomalies and primary omental pregnancy.

## Ethical approval

Ethical Approval is exempted in the case of a case report as per the King Abdulaziz Medical City IRB. Patient anonymity is maintained throughout this manuscript, and consent was obtained for publication from the patient.

## Sources of funding

No funding was provided.

## Author contribution

M.G, S.A.R, A.A, and H.M.R drafted the manuscript. N.F.A.A contributed to reviewing and finalizing the manuscript. All authors reviewed the manuscript for intellectual content and approved the submission.

## Trail register number

1. Name of the registry: Not applicable.
2. Unique Identifying number or registration ID: Not applicable.
3. Hyperlink to your specific registration (must be publicly accessible and will be checked): Not applicable.

## Guarantor

Nuha Fouad Abed Althagafi, MD.

## Consent

Written informed consent was obtained from the patient for publication of this case report and accompanying images. A copy of the written consent is available for review by the Editor-in-Chief of this journal on request.

## Provenance and peer review

Not commissioned, externally peer-reviewed.

## Declaration of competing interest

The authors declare no conflicts of interest.
